# Urinary Extracellular Vesicles as Potential Biomarkers for Urologic Cancers: An Overview of Current Methods and Advances

**DOI:** 10.3390/cancers13071529

**Published:** 2021-03-26

**Authors:** Catarina Lourenço, Vera Constâncio, Rui Henrique, Ângela Carvalho, Carmen Jerónimo

**Affiliations:** 1 i3S-Instituto de Investigação e Inovação em Saúde, Universidade do Porto, Rua Alfredo Allen 208, 4200-135 Porto, Portugal; clourenco@i3s.up.pt (C.L.); angela.carvalho@ineb.up.pt (Â.C.); 2 INEB-Instituto de Engenharia Biomédica, Universidade do Porto, Rua Alfredo Allen 208, 4200-135 Porto, Portugal; 3 IPO Porto Research Center (CBEG CI-IPOP), Cancer Biology and Epigenetics Group, Portuguese Oncology Institute of Porto (IPO Porto), R. Dr. António Bernardino de Almeida, 4200-072 Porto, Portugal; vera.salvado.constancio@ipoporto.min-saude.pt (V.C.); rmhenrique@icbas.up.pt (R.H.); 4 Porto Comprehensive Cancer Center (P.CCC), R. Dr. António Bernardino de Almeida, 4200-072 Porto, Portugal; 5 Department of Pathology, Portuguese Oncology Institute of Porto (IPOP), R. Dr. António Bernardino de Almeida, 4200-072 Porto, Portugal; 6 Department of Pathology and Molecular Immunology, Institute of Biomedical Sciences Abel Salazar, University of Porto (ICBAS-UP), Rua Jorge Viterbo Ferreira 228, 4050-513 Porto, Portugal

**Keywords:** urologic cancers, liquid biopsies, urinary extracellular vesicles, minimally-invasive biomarkers, miRNA and protein biomarkers

## Abstract

**Simple Summary:**

The diagnostic and monitoring techniques used for urologic cancers comprise a group of invasive methodologies that still lack sensitivity and specificity. Therefore, the search for a non-invasive alternative is of extreme importance. Urinary extracellular vesicles are an emerging source of biomarkers that have the potential to be used in cancer detection and management, in a minimally invasive way. However, the increasing interest, allied to the absence of standardization and consensus in strategies to isolate and characterize these vesicles, results in a vast list of candidate biomarkers that present no significant overlap. In this review, we show the variability in the methods implemented to obtain these vesicles and focus on microRNA and protein-derived urinary extracellular vesicles as candidate biomarkers for prostate, bladder and kidney cancers.

**Abstract:**

Urologic cancers are a heterogeneous group of tumors, some of which have poor prognosis. This is partly due to the unavailability of specific and sensitive diagnostic techniques and monitoring tests, ideally non- or minimally invasive. Hence, liquid biopsies are promising tools that have been gaining significant attention over the last decade. Among the different classes of biomarkers that can be isolated from biofluids, urinary extracellular vesicles (uEVs) are a promising low-invasive source of biomarkers, with the potential to improve cancer diagnosis and disease management. Different techniques have been developed to isolate and characterize the cargo of these vesicles; however, no consensus has been reached, challenging the comparison among studies. This results in a vast number of studies portraying an extensive list of uEV-derived candidate biomarkers for urologic cancers, with the potential to improve clinical outcome; however, without significant validation. Herein, we review the current published research on miRNA and protein-derived uEV for prostate, bladder and kidney cancers, focusing on different uEV isolation methods, and its implications for biomarker studies.

## 1. Overview

Urologic cancers are a major cause of morbidity and mortality worldwide, accounting for more than 758,000 deaths in 2018 [1]. Cancers of the prostate, bladder and kidney are the most common urologic cancers, standing among the top 10 causes of cancer-death in European men [2]. Despite the development of novel treatment options in the last decades, many urologic cancer patients endure poor prognosis, due to different but overlapping clinical challenges. For instance, the heterogeneous nature within each cancer type which results from diverse genetic and epigenetic mutations, impairs diagnostic and monitoring efficacy. Moreover, current urologic cancer detection methods are characterized by limited sensitivity or specificity (e.g., serum biomarkers, imaging or urinary cytology) and diagnosis is rendered by means of invasive techniques (e.g., fine-needle aspiration or tissue biopsy), which is associated with a minor, but significant, risk of complications (e.g., bleeding and infection). For instance, prostate cancer is usually diagnosed with image-guided prostate biopsy [3], cystoscopy is commonly used for bladder cancer diagnosis [4] and percutaneous renal tumor biopsies for kidney malignancies [5]. Additionally, clinical diagnosis at early stages is challenging, considering the absence of specific symptoms and the location of the primary tumors. Thus, at diagnosis, patients may already present advanced-stage disease, resulting in poor outcome [6]. Considering the high morbidity and mortality associated with urologic cancers, there is an urgent need for innovative early diagnostic tools and treatment strategies, that might improve clinical outcome and the quality of life of these patients.

Liquid biopsies have been gaining increased attention, due to their minimally invasive nature. This characteristic offers a decrease in morbidity and allows for more frequent collection, enabling a continuous and personalized snapshot of disease evolution. Thus, valuable information concerning tumor burden during treatment and early evidence of recurrence or resistance, due to dynamic tumor monitoring, is within reach. Moreover, unlike tissue biopsies which are obtained from one limited region, that might not even contain the tumor area, liquid biopsy better reflects the phenotypic profile of all tumor subclones present in a patient [7]. Therefore, liquid biopsy is a promising source of non-invasive biomarkers that may help overcome the problems associated with heterogeneity between primary tumors and metastases [8].

Liquid biopsy can be performed in any body fluid and in these fluids there are diverse classes of biomarkers, such as circulating tumor cells (CTCs), proteins, exosomes, cell-free DNA (cfDNA) or other nucleic acids, that may improve cancer detection and prognostication. Although blood has traditionally been the dominant body fluid for cancer biomarkers, such as PSA [9], the interest in urine as a natural and promising source of biomarkers is increasing [10,11]. Urine represents an abundant source of tumor-derived material without background noise. Moreover, besides allowing for non-invasive, repeated and fast collection of samples, urine has additional advantages when it comes to cancers of the urogenital system, since the composition of urine may directly represent the pathophysiological state of the urologic tract [12]. Therefore, urine might be an ideal body fluid for diagnosis and monitoring of patients suffering from urologic cancers.

## 2. Urinary Extracellular Vesicles

Urinary extracellular vesicles (uEVs) have been gaining interest as a class of robust cancer biomarkers since they were discovered in 2004 by Pisitkun et al. [13]. Studies demonstrated that extracellular vesicles (EVs) in urine are secreted by every renal tubule epithelial cell type, as well as podocytes and transitional epithelia from the urinary collecting system [13,14]. Therefore, uEVs provide a suitable starting material for biomarker discovery relevant to a variety of disease processes, including cancer.

EVs are a heterogeneous population of lipid-enclosed structures that can be classified into three main categories: exosomes (30–150 nm diameter), microvesicles (also known as ectosomes, 100 nm–1 µm) and apoptotic bodies (50 nm–5000 nm). This classification is based on the mechanisms by which the membrane vesicles are formed: fusion of multivesicular bodies with the plasma membranes (exosomes), budding of vesicles directly from the plasma membrane (microvesicles) or those shed from dying cells (apoptotic bodies) [15]. Since consensus has not yet emerged about specific markers of EV subtypes and assigning an EV to a particular biogenesis pathway remains extraordinarily difficult, the International Society of Extracellular Vesicles (ISEV) recommends using “extracellular vesicle” as a generic term for all secreted vesicles and urges authors to consider using operational terms that refer to physical characteristics of EVs, such as size (e.g., “small EVs” and “medium/large EVs”), or density; biochemical composition (e.g., CD63+/CD81+ − EVs); or sample and cell of origin descriptions (e.g., uEVs) [16]. Therefore, in this review, the term “EVs” is generally used for extracellular vesicles even if specific EV types are disclosed in the original studies. 

During their formation, EVs incorporate various bioactive molecules from their cell of origin, including membrane receptors, soluble proteins, nucleic acids (mRNAs and microRNAs (miRNAs)) and lipids, which can be transferred to target cells [17]. Besides being intricately involved in intercellular communication, EVs also play a role in cancer progression, through transferring distinct biologically active molecules to local stromal cells and distant organs. Hence, they are thought to generate and sustain a supportive microenvironment that supports primary tumor and metastatic growth [18].

EVs are secreted in nearly all body fluids and present elevated levels in cancer patients relative to healthy subjects [19], making EVs an extremely promising source of cancer diagnostic, predictive and prognostic biomarkers. Furthermore, owing to its encapsulation within membrane vesicles, the biomolecular cargo is stable and protected against exogenous RNases and proteases, even in adverse physical conditions, such as pH extremes, long-term storage, and multiple freeze-thaw cycles, making them an appealing source for robust biomarker development [20,21]. Likewise, understanding of their role in urologic tumorigenesis and tumor progression, coupled with their lipid bilayer membrane also makes them a promising delivery vehicle for therapeutic applications [22,23].

### 2.1. uEV Isolation Methods 

Innovation in science and technology have prompted the constant development of many EV isolation techniques. However, consensus on a single best method has not been reached, yet. Therefore, when planning an EV study, it is of utmost importance to choose the isolation method based on the desired downstream application (protein vs. nucleic acid isolation, biomarker discovery, or functional assays) and from which biological fluid are the EVs going to be extracted (cultured cell media, urine, serum, plasma) [16]. Each technique exploits a particular biophysical or biochemical trait of EVs, such as size, mass density, shape, charge, and surface proteins, to aid their isolation. Generally, ultracentrifugation (UC) and density-gradient ultracentrifugation (dUC) are the most frequently used isolation methods [24]. Indeed, the EV-TRACK database, assessed in January 2021, using the keywords “urine” and “Homo sapiens”, revealed that UC is the most used method for isolation of uEVs. Interestingly, UC is included in nine out of the 10 most used methods. Addition of filtration and density gradient to UC are the second and third isolation choices, respectively, with isolation kits such as ExoQuick Exosome Precipitation Solution (System Biosciences, Palo Alto, CA, USA) filling the last spots in the top 10 [25]. A summary of studies comparing uEVs isolations methods is depicted in Table 1.

#### 2.1.1. Density-Based Methods

- Ultracentrifugation

UC uses the different size and density of EVs from other components in the sample to isolate the vesicles. This method comprises three centrifugation steps: a first low-speed centrifugation (300–500× *g*, 5–10 min) that eliminates cells and cell debris; a second medium speed centrifugation (10,000–20,000× *g*, 10–20 min) that eliminates larger vesicles; and high-speed centrifugations (100,000–200,000× *g*, 1–3 h) to extract EVs [36]. This method has been broadly used for proteomic and transcriptomic analysis of uEVs and EVs in general, since it provides EVs with high purity, is easy to use and requires very little technical expertise. However, it is a time-consuming technique, with low sample throughput, and is not practical for analysis of large clinical cohorts. Furthermore, EVs might be ruptured during UC; therefore, EV recovery is low due to sample loss. Nevertheless, in several comparative studies, UC often presents overall advantages and is the chosen method for downstream applications [27,31,32].

- Density Gradient Centrifugation

By adding a density gradient centrifugation to UC, using sucrose or iodixanol (OptiPrep™, AXIS–SHIELD, Oslo, Norway), the purity of EVs is increased [37]. A reduced volume of the sample is loaded on a density gradient medium, in a centrifuge tube. Samples are centrifuged, and the density decreases progressively from the bottom to the top. The EVs travel through the gradient until they reach the point at which their density matches the one of the surrounding solutions [36]. This method presents highly pure EVs and is a relatively easy to perform technique; however, since the separation is based on density, the EV fraction may contain other vesicles of different origins but similar densities. Additionally, dUC is not suitable for analysis of large numbers of clinical samples when samples may require long-running times to reach equilibrium (i.e., low throughput). Furthermore, compared to UC, the protein yield is lower [38].

#### 2.1.2. Size-Based Techniques

- Ultrafiltration (UF)

EVs can also be isolated based on their size, using nanomembrane concentrators. Before UF, samples are filtered through a 0.22 µm filter to remove larger microvesicles and apoptotic bodies. The soluble components are then passed through a filter with a molecular weight cut-off of 3–100 kDa. This approach, together with short periods of centrifugations have been shown to rapidly enrich for uEVs as effectively as UC and does not require special equipment [39,40].

Unfortunately, besides EVs, this method retains and concentrates soluble proteins that are present in urine, decreasing EV yield. Therefore, and although some studies have shown that UF is preferred to UC [24,29,33] and extracts the highest EV RNA yield from the urine of healthy individuals [29], nanomembrane UF does not seem an efficient method to isolate uEVs from the urine of patients with urologic diseases. These pathologies usually present a high concentration of soluble proteins present in urine that obstruct the nanomembrane during UF [27]. This results in reduced efficiency and the presence of soluble proteins in sufficient quantity to interfere with the detection of less abundant uEV cargo.

- Size Exclusion Chromatography (SEC)

SEC is a column-based technique that also uses EV size properties to isolate them from the rest of the sample. A porous stationary phase sorts out macromolecules, vesicles and soluble proteins according to their size. The pore size is determined by the choice of the exclusion matrix, for instance, Sepharose 2B is commonly used for EV isolation [32,41]. SEC results in a high-purity fraction of EVs and retains structural integrity and biological activity of the EVs. The major co-isolated non-EV components are particles above the size cut-off, which may include viruses and protein aggregates. Interestingly, SEC was used after UC to improve the purity of uEVs from nephrotic syndrome patients and showed low protein contamination [27]. 

SEC enrichment is relatively inexpensive with a high-throughput, which makes SEC applicable for large-scale analysis. Moreover, SEC could be partially automatized and adapted for diagnostic and monitoring labs. Although chromatographic isolation increases the purity of the yield, it does not reduce the processing time and requires specialized equipment [42].

- Polymer Precipitation

Polymer precipitation captures EVs at low speeds of centrifugation in combination with polymers. The most commonly used polymer is polyethylene glycol (PEG) and ExoQuick Exosome Precipitation Solution (System Biosciences, Palo Alto, CA, USA) is a popular commercial kit for EV precipitation [36]. Samples are usually pre-cleared of large cell fragments and cell debris by low-speed centrifugation and are incubated with polymers for 15 min to 12 h, depending on the polymers used. EVs are then enriched by either low-speed centrifugation or filtration. This is an easy-to-use method, does not require specific equipment and is flexible with sample volume. It was shown that uEV precipitation achieves the highest yield compared to UC and UF [28]. The highest quantities of miRNAs were extracted for subsequent profiling analysis, but these results were achieved with the addition of DTT and protocol modifications. Moreover, a significant disadvantage of this method is the co-precipitation of abundant non-EV contaminants, such as proteins and polymeric materials [28,43,44].

#### 2.1.3. Microfluidic-Based Strategies

Although different uEV isolation methods are currently available, the variety and technical complexity of some of the proposed methods limits the comprehensive exploration of EV cargo with adequate yield and purity to allow for in-depth protein and miRNA profiling [43]. Hence, there is an urgent need to establish simple, high-throughput and rapid methods for isolating disease-specific EVs [44]. Current methods and/or commercial kits are time-consuming, expensive, and disease-specific EVs are not specifically isolated. Alternatively, microfluidic-based technology shows great promise, depicting better EV purity, higher recovery rates, lower costs, and decreased isolation times [45,46,47]. Therefore, the direct isolation of a pure population of uEVs, using specific surface markers that allow for uEVs characterization and further applications would be of great interest for discovery of non-invasive biomarkers for urologic cancers.

Several microfluidics-based devices have been developed for isolation of EVs from cell culture medium [48], serum [49] or plasma [50]. However, studies using urine samples are scarce. ExoTIC (exosome total isolation chip) is a simple, easy-to-use, modular device that presents high-yield and high-purity EV isolation from a variety of biofluids, including urine. This chip achieved an ∼4−1000-fold higher EV yield, compared to UC [46].

Regarding urine samples from urologic cancers, Liang et al. developed an integrated double-filtration microfluidic device that isolated and enriched uEVs from BlCa patients and subsequently quantified the EVs via a microchip ELISA. They reported a higher concentration of uEVs in BlCa patients compared to healthy controls [19]. Exodisc is a lab-on-a-disc device integrated with two nanofilters, where BlCa patient urine samples’ were automatedly enriched in uEVs within 30 min using a tabletop-sized centrifugal microfluidic system [51].

These results suggest that applying microfluidic-based methods may be useful in clinical settings to test uEV-based biomarkers for urologic cancer diagnostics.

### 2.2. Characterization Methods

Similar to isolation methods, the broad interest and complexity of EVs resulted in the development and implementation of a large variety of techniques to characterize these vesicles. However, no single technology seems capable of fulfilling the full spectrum of EV properties. Furthermore, EV heterogeneity is reflected in a broad distribution of biochemical and physical properties. Therefore, according to MISEV2018 guidelines, EV detection and characterization are recommended to be assessed by multiple and complementary techniques, to ensure that biomarkers are associated with EVs and not contaminants [16].

Firstly, it is recommended that EVs are described quantitatively according to their source (e.g., volume of urine), followed be quantification of particles (e.g., nanoparticle tracking analysis (NTA), transmission electron microscopy (TEM)) and/or total protein amount (e.g., Bradford). As for physical properties of EVs, electron microscopy is the most direct method to determine the structure and intactness of individual EVs. However, traditional electron microscopy includes dehydration and fixation treatments that may cause shrinking of EVs and therefore alter its morphology [52]. This can be avoided by cryo-electron microscopy. Sample’s rapid freezing better preserves the morphology of vesicles and can therefore be more suitable for EV studies [53,54]. However, this technique requires highly sophisticated equipment and technical expertise.

NTA uses the Brownian motion to quantify the number and size distribution of EVs. By analyzing the trajectory of a particle in a static solution, it is possible to estimate the diffusion coefficient and size of individual vesicles [55]. If many trajectories are assessed, the concentration and size distribution of EVs can be estimated in a sample. However, short measured trajectories of in and out of focus vesicles and aggregates, that may lead to misrepresentation of particle concentration, are some limitations of this technique [56]. 

After quantification, EVs may be characterized regarding the presence of specific molecules (e.g., immunoblotting). Immunoblotting characterizes the biochemical aspect of EVs. This technique uses specific EV marker proteins to show the purity and enrichment of the vesicle portion of the samples. After lysis, proteins are released from the EVs and can be quantified by dot blot or western blot assay. CD9, CD63, ALIX or Tsg101 are commonly used specific EV-associated proteins, that confirm the presence of EVs in the sample. This is a fast and simple detection method; however, it is only semi-quantitative, does not provide the content information of an individual EV and does not recognize the heterogeneity of EV populations [16].

Lastly, and to better ensure the purity of the EV preparation, co-isolated molecules must be analyzed (e.g., Tamm–Horsfall protein (THP) in urine) [16]. Although immunoblotting, TEM and NTA are the most used techniques for EV characterization [57], other technologies such as nanoscale flow cytometry, ELISA and dynamic light scattering (DLS) are also included in the wide range of approaches that are under rapid development and will help improve EV characterization. By combining some of the referred methods, good EV characterization can be accomplished [58].

## 3. uEVs as Biomarkers in The Three Urologic Cancers: miRNA and Protein Markers

The potential of uEV-derived biomarkers for improving the clinical outcome of urologic cancer patients has potentiated intensive research. Despite these efforts, no biomarker is currently implemented in urologic oncology. Any of the contents of EVs (proteins, nucleic acids and lipids) can be studied for prostate, bladder or kidney cancers; however, proteins and miRNA are among the most investigated EV-cargo types [59]. Proteins have been extensively studied as free molecules in urine, and the comparison of its proteome with EV-derived proteins is of great interest. Indeed, in one comparative study, Lee et al. concluded that uEVs might be a good source for proteomic analysis owing to lower levels of highly abundant “contaminant” proteins like albumin [60]. On the other hand, the focus on miRNAs is due to the fact that they represent the largest component of EV-content and their role in cell-cell communication.

### 3.1. Prostate Cancer

Prostate cancer (PCa) is the second most frequent cancer and the fifth leading cause of cancer death in men [1]. PCa has a complex etiology meaning that accurate diagnosis and targeted treatment remains challenging [61,62]. Despite low specificity, prostate-specific antigen (PSA) is routinely used for PCa detection. However, its inability to discriminate between indolent and aggressive cancers causes overdiagnosis and overtreatment [63]. Following detection of raised serum PSA levels, patients are subjected to invasive prostate biopsy to histologically confirm the presence of PCa. As a consequence, about 70–80% of the prostate-tissue biopsies are deemed unnecessary [64]. Furthermore, due to the multifocal nature of PCa, there is a possibility that the cancer focus detected is not the most clinically significant. This is further compounded by the possibility of the biopsy missing cancer foci, resulting in a false negative result. Thus, finding minimally-invasive sources of biomarkers that improve PCa detection and perfect clinical decision-making is of utmost importance. Indeed, after detection of PCa at an early stage, discrimination of aggressive from indolent tumors, improvement of prognostic evaluation in localized and metastatic tumors, as well as evaluation of treatment response and development of resistance, are major clinical challenges. Remarkably, studies indicate that PCa-derived uEVs may mediate disease progression and be used to monitor PCa patients (Table 2) [65].

#### 3.1.1. Protein Biomarkers in PCa

Mass spectrometry and immune-based and targeted proteomics have allowed for identification of thousands of proteins encapsulated within uEVs, that may be used as biomarkers for PCa diagnosis [35,68,72]. UC isolated uEV proteins disclosed potential for full differentiation of PCa patients from non-disease controls. For instance, TM256 in combination with LAMTOR1, highly-specific in PCa patient samples, increased sensitivity to 100% [66]. A combination of flotillin 2 and Parkinson protein 7 in uEV could also differentiate between PCa patients and healthy subjects with 68% sensitivity and 93% specificity [70]. Even though Overbye et al. and Wang et al. used similar cohorts, urine preparation and uEV isolation methods, these studies did not reach the same candidate biomarkers, although both detected an increase of the same proteins in PCa patients.

Dhondt et al. used another strategy to isolate uEVs. Bottom-up Optiprep density gradient centrifugation separated uEVs with high specificity and repeatability, presenting minimal THP and soluble protein contamination. Furthermore, differential quantitative proteomic analysis of patients with PCa and benign prostatic hyperplasia (BPH) showed a significant decrease in FKBP5, FAM129A, RAB27A, FASN, NEFH proteins after local PCa treatment. Moreover, proteomic analysis of uEV from BlCa and RCC patients disclosed specific protein signatures, reflecting their cancer tissues of origin [35].

Some studies have also explored the role of uEV-derived proteins as prognostic biomarkers for PCa. Sequeiros et al. isolated uEVs from low- and high-grade PCa patients using UC and reported that the ADSV-TGM4 panel accurately classified non-PCa and PCa patients and a panel of five proteins (PSA; CD63 antigen, CD63; putative glycerol kinase 5, GLPK5; N-sulphoglucosamine sulphohydrolase, SPHM; and prostatic acid phosphatase, PAPP) significantly discriminated between high- and low-grade PCa [69]. FABP5 might also potentially be used as a biomarker to predict or confirm the presence of high-Gleason score (GS) PCa before prostatectomy [68].

Welton et al. used a combination of UC and SEC to isolate urinary vesicles from two groups of metastatic PCa patients: newly diagnosed (before receiving any therapeutic interventions) or having failed all therapeutics (bearing progressive disease). Remarkably, several proteins, such as Afamin, cardiotrophin-1, CDON, were found increased in progressive disease, highlighting the potential to identify treatment failure [67].

#### 3.1.2. miRNA Biomarkers in PCa

miRNA-derived uEVs have also been a target for numerous diagnostic biomarker studies in PCa. miR-19b achieved 100% specificity and 95% sensitivity in discriminating cancer patients from healthy individuals [72], whereas Koppers-Lilac et al. showed that miRNA isoforms (isomiRs) of miR-21, miR-204 and miR-375 with 3′ end modifications were highly discriminatory between controls and PCa patients [71]. Davey et al. also identified two miRNAs (miR-375-3p and miR-574-3p) allowing for discrimination of PCa patients from healthy individuals among cancer suspects submitted to prostate biopsy, with uEVs isolated by an affinity method using the Vn96 peptide [78]. On the other hand, miR-196a-5p and miR-501-3p were downregulated in PCa samples [75].

Foj et al. reported that miR-21, miR-375, and let-7c were significantly upregulated in the PCa patients compared with healthy donors, and let-7c levels were also significantly associated with clinical stage [76]. Xu et al. searched for an easy and inexpensive method to enrich EVs from urine samples, enabling differential expression of four PCa-related miRNAs (miR-572, miR-1290, miR-141, and miR-145). UEVs isolated by hydrostatic filtration dialysis (HFD) method from patients with PCa, BPH and healthy individuals, depicted an overall performance similar to UC. The levels of miR-145 in uEVs were significantly increased in patients with PCa and a significant increase was also observed in patients with GS ≥ 8 PCa compared with GS ≤ 7 [73]. UEV miR-2909 recruitment may provide a potential non-invasive candidate diagnostic marker for the detection of PCa and characterization of its aggressiveness. This miRNA was conspicuously expressed in PCa subjects compared to BlCa patients and showed characteristic variation as a function of PCa aggressiveness compared to serum PSA. Interestingly, this study used a precipitation commercial kit (Exiqon miRCURY exosome isolation kit) to isolate EVs from urine [74].

### 3.2. Bladder Cancer

Bladder cancer (BlCa) is the tenth most common type of cancer, and the second most common urologic malignancy worldwide [1]. About 70% of all newly diagnosed cases are non-muscle-invasive (NMIBC), comprising a heterogeneous group of patients with Ta and T1 papillary tumors as well as urothelial carcinoma in situ (CIS). The recurrence rate ranges from 50–70%, and roughly 10–20% of NMIBC will progress to muscle-invasive bladder cancer (MIBC) [79]. MIBC presents a 50% risk of developing distant metastases in the first two years [80], and a reported five-year survival rate of 40–60% [81]. Hence, after initial treatment, patients are committed to lifelong surveillance to early identify recurrence and prevent progression into invasive disease. Due to its high recurrence rate, BlCa is considered the most expensive type of cancer, since current monitoring tools rely on cystoscopy, an invasive, costly and uncomfortable procedure [82]. Although multiple urine-based tests are commercially available, their sensitivity, specificity and diagnostic accuracy remain suboptimal and are; therefore, of limited clinical usefulness [83]. Hence, the development of new strategies to identify biomarkers for early diagnosis and monitoring is of utmost importance. Due to its location and function, bladder-derived EVs are directly released in urine, making BlCa the malignancy that may benefit more from using urine as a biofluid. Therefore, more extensive data about uEV-derived proteins and miRNAs (Table 3) has been published for BlCa, compared to other urologic cancers [84].

#### 3.2.1. Protein Biomarkers in BlCa

Using UC, several proteins were identified as candidate BlCa biomarkers due to their enrichment in patient uEVs compared to healthy volunteers [85,89].

Chen et al. identified seven proteins differentially enriched in low- vs high-grade BlCa (i.e., APOA1, CD5L, FGA, FGB, FGG, HPR and HP). Finally, ELISA quantified tumor-associated calcium-signal transducer 2 (TACSTD2) and confirmed its potential value for diagnosis of BlCa [86]. Lee et al. showed that 56 proteins were significantly increased in BlCa urine, including proteins for which expression levels varied according to cancer stage [60]. Through dUC, Lin et al. isolated uEVs from BlCa patients. UEV proteins alpha 1-antitrypsin and histone H2B1K could facilitate rapid diagnosis and prognostication of BlCa [88]. Hiltbrunner et al. aimed to investigate if pro-carcinogenic EVs could be detected in urine from histologically down-staged BlCa patients. They discovered that 40 proteins were significantly overexpressed in bladder uEVs, including known oncogenes such as TPP1, TMPRSS2, FOLR1, RALB and RAB35, whereas SLC4A1 disclosed lower expression. Although patients were histologically tumor-free at cystectomy, bladder urine contained EVs with a carcinogenic metabolic profile. This suggests a continuous release of EVs from the bladder, which may promote recurrence at distant sites through metabolic rewiring, even after apparent complete downstaging, encouraging of cystectomy even in completely down-staged patients [90].

#### 3.2.2. miRNA Biomarkers in BlCa

While analyzing miRNAs from matched formalin-fixed paraffin-embedded (FFPE) with other biofluids, Armstrong et al. discovered that a significant number of miRNAs enriched in tumors, uEVs and WBCs were not enriched in plasma EVs. These data suggest that different biofluids may harbor different and overlapping biomarker populations and therefore are worth exploring in larger cohorts of patients. uEV miRNA profiles, isolated with Norgen Urine Exosome RNA Isolation Kit, were compared with matched BlCa tissues and showed that miR-205, miR-200c-3p and miR-29b-3p were common to both tumor tissues and uEVs. This demonstrates that, as suggested, EVs reflect the molecular signature of parental tumor cells and might; therefore, serve as a valid tools for molecular characterization of tumors themselves [91].

De Long et al., isolated EV-derived RNA via UC and a panel of four miRNAs (miR-21, miR-93, miR-200c and miR-940) presented different expression levels between BlCa and cancer-free patients, disclosing a sensitivity of 88% and specificity of 78%, providing evidence that miRNA profiling in cell-free urine holds promise for the development of valuable clinical diagnostic tools [92]. Matsukazi et al. also extracted miRNA from uEVs by UC and used a microarray that identified five miRNAs (miR-155-5p, miR-15a-5p, miR-21-5p, miR-132-3p and miR-31-5p) overexpressed in uEVs from BlCa patients compared to healthy volunteers. Remarkably, miR-21-5p was the most promising biomarker, being also overexpressed in uEVs from BlCa patients with negative urine cytology [93]. 

Andreu et al. isolated uEVs by UC and their miRNA composition were evaluated. Real-time PCR analysis pointed to miR-375 and miR-146a as diagnostic markers of high-grade and low-grade BlCa, respectively [87].

Another challenge in BlCa is using urine samples to identify patients with MIBC to select patients for radical surgical treatment, as currently no markers specifically detect MIBC. Therefore, Baumghart et al. isolated uEV using a commercially available kit, and also by comparing with FFPE tumor tissues, showed that miR-146b-5p and miR-155-5p in uEVs might serve as biomarkers to distinguish MIBC from NMIBC [94].

### 3.3. Kidney Cancer

Kidney cancer is the fifth most common cancer in European men [2]. Renal cell carcinoma (RCC) is the most frequent type of kidney cancer and one of the most common urologic cancers, approximately representing 90% of all kidney malignancies [95]. It represents 2.2% of all malignancies and is responsible for about 2% of all cancer-related deaths [1]. Among RCC, clear-cell type (ccRCC) represents about 75% of cases [96]. Although most RCC cases are currently identified by means of medical imaging, confirmation of diagnosis requires a biopsy [97]. However, this is an invasive procedure of limited repeatability and success rate [41,98]. Another challenge, particularly in small renal masses (SRMs), is the discrimination between benign and malignant lesions, which is key to decide whether surgery might be necessary [99]. Importantly, RCC diagnosis is often incidental since many RCC remain asymptomatic until late disease stages. Therefore, RCC diagnosis is often delayed until the disease is advanced, with 30% of patients harboring metastasis at the time of diagnosis and with another 30% developing metastasis during the course of the disease [100]. It is not surprising, hence, that RCC discloses the highest mortality rate among genitourinary cancers. Moreover, nephrectomy remains the most effective treatment [101], since RCC is resistant to both chemotherapy and radiotherapy [102], although it is only curative for localized disease. Therefore, development of alternative diagnostic tools is of major clinical interest. Compared to BlCa and PCa, fewer studies have addressed the potential use of uEVs as diagnostic tool for RCC (Table 4), although, the use of uEV as biomarkers and therapeutic options has been well explored for other renal pathological conditions [103].

#### 3.3.1. Protein Biomarkers in RCC

Raimondo et al. remains the only study establishing a uEV-protein profile of RCC patients compared to control subjects. Vesicles were isolated by UC and proteomic analysis led to the identification of 186 proteins from RCC patients. Ten proteins were selected and validated by western blot. This study showed, for the first time, that RCC-derived uEVs have a protein profile different from the ones derived from healthy individuals, suggesting that protein uEVs might provide a tool for identifying RCC new biomarkers [104].

#### 3.3.2. miRNA Biomarkers in RCC

Global uEV-miRNA expression from patients with ccRCC was assessed by Butz et al., with NORGEN isolated uEVs. Although uEVs were not characterized in this study, different miRNA combinations, including miR-126-3p, miR-449a, miR-486-5p and miR-34b-5p, were able to discriminate ccRCC patients from healthy controls, and also distinguish SRMs and benign tumors from healthy controls. This data is very promising and suggests that the identified urinary miRNAs may serve as diagnostic biomarkers, improving decision making for RCC patients [105]. Song et al. also identified differentially expressed miRNAs from UC-isolated uEVs of ccRCC patients. miR-30c-5p levels significantly differed between ccRCC patients and healthy controls, constituting a potential diagnostic biomarker for early-stage ccRCC [106].

Although promising, further studies are needed to expand and implement the use of uEVs as a tool for the identification of new RCC biomarkers.

## 4. Limitations of Urine as a Biofluid

Urine presents several advantages as a biofluid for discovery and implementation of new biomarkers for urologic cancers. For instance, urine has less concentration of non-EV proteins than plasma, it is relatively fast and cost-efficient to collect compared with other biofluids and is in direct contact with cells from the urinary tract. However, urine presents special properties and characteristics that require special treatment for isolation of EVs, that result in highly variable protocols (Table 1). Urine is a dynamic biofluid that depicts wide ranges of pH, osmolality, protein concentration and composition of dispersed solutes, even within the same individual and permanency time within the bladder. This variable nature adds to the complexity of downstream EV isolation protocols. Therefore, in an attempt to normalize methods for collection, storage, and preservation of uEV, Zhou et al. concluded that protease inhibitors (PI) must be added to urine for protein preservation. They also stored urine at different temperatures and reported that storage at −80 °C with extensive vortexing after thawing maximizes the recovery of uEVs. uEVs also remained intact during long-term storage. Furthermore, there was no significant difference in EV-associated protein between first and second morning-collected urine [107]. 

Another obstacle in uEV isolation and purity, is the presence of contaminants, in case of hematuria and/or proteinuria. Hematuria is a condition commonly present in renal and urological diseases, which was shown to alter EV yield and cargo profile. Interestingly, trypsin can be added to samples to reduce hematuria effects [108]. THP is the most abundant protein in urine and forms aggregates that retain EVs, decreasing its yield. Therefore, treatment with dithiothreitol (DTT) or 3-((3-cholamidopropyl)-dimethylammonio)-1-propanesulfonate (CHAPS), have been proposed to release uEVs from their complex with THP. Treatment with DTT is the most widespread method, with studies showing improved recovery of uEVs [26,28,32] and decreased contamination in extracted miRNA samples [31]. However, THP monomers may remain in the isolated EV fraction and interfere with further analysis. In addition, DTT may alter the native structure of proteins and their complexes on EV surface by reducing disulfide bonds, which may also influence the results of EV proteomic analysis. Thus, urine treatment with DTT is not always effective [109]. Furthermore, pre-treatment of urine by serial centrifugation is recommended, to remove urinary sediment, including whole cells, large membrane fragments and other debris. DTT, PI and filtration of samples before or during isolation, may also be added to the process since urine contaminants like THP and albumin can be co-isolated with EVs [26,32]. In addition, although most studies use density-based techniques for uEV isolation, these methods depend on multiple variables such as force, time and temperature of centrifugation (Table 1). The addition of all these factors results in a highly variable and complex outcome that makes difficult the comparison between uEV studies.

In addition, several studies on PCa collected urine after prostatic massage, since this procedure seemed to increase the amount of EVs isolated in urine. However, contrarily to these studies, Overbye et al. identified possible diagnostic biomarkers when urine was collected directly, without previous prostatic massage, to facilitate the use of potential EV-based PCa markers in the clinics [66].

## 5. Discussion

The interest of studying uEV content for the discovery of novel biomarkers for urologic cancers is easily explained by their potential role in cancer diagnosis, prognosis and monitoring, as well as their putative therapeutic applications. Moreover, uEVs disclose a non-invasive and safer alternative to the currently available diagnostic and monitoring procedures for urologic cancers.

**Figure 1:**
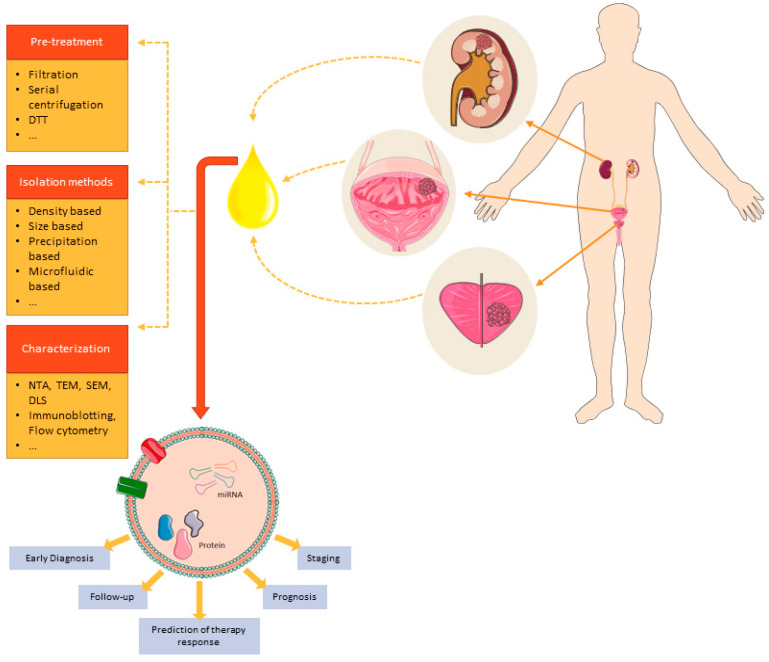
Overview of processing steps for uEVs-derived miRNA and protein biomarkers for urologic cancers.

Despite the relatively large number of studies focusing on the discovery of uEV-based biomarkers, EV cargo research is still at an early stage and no gold standard workflow has been established (Figure 1). Furthermore, there is an extremely low overlap of candidate biomarkers across published studies (Table 2, Table 3 and Table 4). This may be due to differences in experimental design and the composition and size of patient cohorts used in the discovery experiments. 

Nevertheless, although additional studies are needed to prove the clinically utility of these markers, there is potential in the overall biomarker candidates, since noteworthy sensitivities and specificities have been reported. Interestingly, other strategies such as combination of different classes of biomarkers, might improve biomarker performance. For instance, Davey et al. also evaluated a mRNA panel and showed that a combination of mRNAs and miRNAs increased sensitivity and specificity, compared with the individual panels. Moreover, when clinical characteristics and PCA3 levels were added, an AUC of 0.96 was achieved [78]. Xu et al. also observed improved discrimination between PCa and BHP when uEV-derived miR-145 was associated with serum PSA [73].

Currently, there is still limited knowledge about the function and molecular machinery for differentiating between subtypes of EVs [16]. However, because different subtypes might express different biomarkers, a clear characterization should be also reported. Although both terms “EVs” and “exosomes” were applied in the several papers reviewed herein, the majority of those with size characterization could be included in the class of “small EVs” (according to MISEV 2018 guidelines). However, in many of those studies, this type of data was not clear, what may difficult advances in biomarker discovery and validation.

Therefore, standardization of approaches for isolation and characterization of uEVs and analysis of its cargo is urgently needed to enhance implementation of these candidate biomarkers in clinical practice. Importantly, easy to perform EV isolation methods are lacking and hamper its translation for clinical use.

Studies comparing methods of isolation are mostly performed using healthy samples. These studies often use different methods and characterize EVs to select the best option for downstream applications. However, in cancer biomarker studies, most studies opt for only one method and hardly perform a thorough characterization of the isolated particles, as seen by comparing the studies of Table 1 with Table 2, Table 3 and Table 4. On other hand, methods used to isolate EVs from urine from a healthy individual might not necessarily be viable to isolate EVs from the urine of a patient with urologic disease, owing to the nonspecific association of highly abundant soluble proteins [27]. Therefore, more studies in urologic malignancies comparing different isolation methods should be carried out, due to the complexity of urine and how that complexity may change the performance and results of the technique. Moreover, even within the same approach for uEV isolation, there are a wide diversity of protocols that compromises the verification, comparison and analysis of the data obtained in different studies.

The ideal uEV isolation method for clinical use should be a simple and inexpensive technique that does not require complex equipment. Moreover, it should be fast and allow for the isolation of high quantity of EVs. Although a universal EV isolation method might not be possible, further research and standardization of currently available methods might help in the discovery and approval of different types of biomarkers.

## 6. Concluding Remarks and Future Perspectives

Over the last decade, research of uEV cargo has shown the potential of urine-based biomarkers for kidney, bladder and prostate cancer management. The biggest challenges for progress are the lack of standardization on urine sampling and processing and lack of highly effective and robust methods for reproducible and fast uEV isolation. This heterogeneity in sampling and procedures hampers the implementation of potential uEV-biomarkers in clinical practice. A consensus must be reached for future uEV miRNA and protein biomarker studies to be performed in a properly selected, large, clinical sample cohorts and its further validation in multicenter prospective studies.

Additionally, developments in other techniques, such as NGS, microfluidic-based uEV isolation methods and advanced MS-based proteomics, may be powerful allies in paving the way for new approaches in discovery and clinical implementation of uEV biomarkers in cancers of the urologic tract.

Thus, major efforts have been made to implement new guidelines for standardizations in sample throughput, uEV isolation and characterization methods, and analysis of EV content. Implementation of ISEV suggested protocols and steps are part of a strategy that will improve the progress in this field and potentiate the discovery of specific and sensitive biomarkers with clinical importance in urologic malignancies diagnosis and monitoring.

## Figures and Tables

**Table 1:** Studies comparing different urinary extracellular vesicles (uEV) isolation methods.

Study	Urine Source (*n*)	Type of Urine/Pre-Treatment	Isolation Methods	Description	Characterization
[26]	Healthy (10)	First-void urine and 12 h collection	UC	**Centrifuged** (17,000× *g*, 10 min, 37 °C); **IS**; **centrifuged** (17,000× *g*, 10 min, 37 °C); **centrifuged** (200,000× *g*, 1 h, 37 °C)	TEM WB: Alix, TSG101, CD9, HSP70, AQP2
		**PI**	UC + DTT (200,000× *g*)	Described as above; **DTT**	
			* UC + DTT (17,000× *g*)	**Centrifuged** and **IS**, as described for UC; **DTT**; **centrifuged** (17,000× *g*, 10 min, 37 °C); **centrifuged** (200,000× *g*, 1 h, 37 °C)	
[27]	Healthy (NS) IMN (NS) FSG (NS)	NS	UC	**Centrifuged** (200,000× *g*, 110 min); **IS**	TEM WB: AQP2, neprilysin, PODXL and albumin
			UC + DTT	Described as UC; **DTT**; **centrifuged** (200,000× *g*, 110 min)	
		**Filtered**; **SC** (17,000× *g*, 15 min)	UF	**Vivaspin 20NC** (100 kDa MWCO); **centrifuged** (3000× *g*); pre-heated Laemmli buffer	
			* UC + SEC	**Centrifuged** (200,000× *g*, 110 min); **IS**; **SEC** **column**; **centrifuged** (3000× *g*), Amicon **Ultra-4** (10 kDa MWCO)	
[28]	Healthy (4)	First-void urine	* UC	**Centrifuged** (17,000× *g*, 10 min, 37 °C); **IS**; **DTT**; **centrifuged** (17,000× *g*, 10 min, 37 °C); **centrifuged** (165,000× *g*, 70 min, 37 °C); IS; DTT; **centrifuged** (165,000× *g*, 70 min, 37 °C)	ELISA: CD9 WB: Alix and TSG101
		**PI**; different pre-process depending on the isolation method	* dUC (sucrose cushion)	Steps prior to last high-speed centrifugation as described above; **30**% **sucrose**/**D_2_O**; **centrifuged** **2×** (165,000× *g*, 1 h, 37 °C)	
			UC + 0.22-µm filter	**1st Centrifuation**, **IS**, **DTT** as described above; **centrifuged** (17,000× *g*, 10 min, 37 °C) + **0.22** **µm** **filter**; **centrifuged** (165,000× *g*, 70 min, 37 °C); **IS**; **DTT**; **centrifuged** (165,000× *g*, 70 min, 37 °C)	
			UF	Steps prior to last high-speed centrifuged as described in UC; **Vivaspin** **20** **NC** (100 kDa MWCO); **centrifuged** (3000× *g*, 1 h, 25 °C); **concentrator**	
			Precipitation (ExoQuick std)	**Centrifuged** (3000× *g*, 10 min, 25 °C); **ExoQuick-TC** (4 °C, >12 h); **centrifuged** (1500× *g*, 30 min, 25 °C)	
			* Precipitation (ExoQuick modified)	**1st Centrifugation**; **IS**; **DTT**; **2nd centrifugation** as above; **ExoQuick-TC**; **centrifuged** (10,000× *g*, 30 min, 25 °C)	
^#^ [24]	ADPKD (7) Healthy (7)	First-void urine	UC	**Centrifuged** (110,000× *g*, 17 °C, 2.5 h); **concentrated** (100 kDa filter)	IZON qNano TEM WB: CD24, AQP2
		**PI**; **centrifuged** (1800× *g*, 10 min) UC, UF → **−80** **°C**; heated to 40 °C; **centrifuged** (17,000× *g*, 20 °C, 15 min)	UF	**Filtered** (100 kDa MWCO); **centrifuged** (1500× *g*, 20 °C); **spin** **device**	
			dUC	**Centrifuged** (4000× *g*); **centrifuged** (150,000× *g*, 1 h); 5–**30**% **sucrose**/**D_2_O**; **centrifuged** (100,000× *g*, 24 h); **fractionation** **device**	
[29]	Healthy (NS)	First-void urine	* UF	**Centrifuged** (20,000× *g*, 20 min); **Vivaspin** **20** **NC** (100 kDa MWCO); **centrifuged** (3500× *g*, 1 h); **DTT**	DLS WB: AQP2
		**PI**; **centrifuged** (3500× *g*, 40 min); **filtered** (0.22 μm); **−20** **°C**	UC	**Centrifuged** (20,000× *g*, 20 min); **centrifuged** (200,000× *g*, 1 h); DTT; **centrifuged** (200,000× *g*, 1 h)	
			Precipitation (ExoQuick)	**ExoQuick-TC** (4 °C, >12 h); **centrifuged** (1500× *g*, 30 min)	
[30]	Healthy (10)	First-void urine	UC	**Centrifuged** (100,000× *g*, 90 min); **ERB**	TEM WB: CD9, CD10, CD63, TSG101, CD10, Alix, AQP2 and FLT1
			Precipitation (ExoQuick)	**ExoQuick-TC** (4 °C, 16 h); **centrifuged** (1500× *g*, 30 min); **ERB**	
		**Centrifuged** (2000× *g*, 10 min); **filtered** (0.22-µm); **−80** **°C**	TEI solution (INVITROGEN)	**INVITROGEN mix** (1 h, RT); **centrifuged** (10,000× *g*, 1 h); **ERB**	
			* Norgen (modified)	**Slurry component**; **centrifuged** (2000× *g*, 2 min)	
			Lectin-based purification (STL)	Biotinylated STL/Streptavidin Dynabeads (1 h, RT)	
[31]	Healthy (NS)	NS	* UC	**Centrifuged** (2000× *g*, 15 min and 10,000× *g*, 30 min); **DTT**; **centrifuged** (17,000× *g*, 10 min, 37 °C); **centrifuged** **2×** (100,000× *g*, 1.5 h); **PI**	TEM WB: Alix and TSG101, CD63
		−**80** **°C**	Norgen (modified)	Remove debris; **slurry** **component**; **centrifuged** (15,000× *g*)	
			TEI solution (INVITROGEN)	Remove debris; **INVITROGEN** **mix**; **centrifuged** (15,000× *g*)	
[19]	BlCa (16) Healthy (8)	NS	UC	**Centrifuged** (100,000× *g*, 70 min); **−80** **°C**	TEM DLS Fluorescence staining: CD9
		**Centrifuged** (20,000× *g*, RT, 15 min); **filtered** (0.22 μm)	* Double-filtration microfluidic	**Double-filtration** **device**	
[32]	Healthy (5)	First-void mid-stream urine	UC	**Centrifuged 2×** (120,000× *g*, 70 min)	TEM NTA WB: CD63, CD9, TSG101 and CD81
			PEG	**PEG solution** (4 °C, 12 h); **centrifuged** (1000× *g*, 30 min)	
		**Vortexed**; **SC** (200× *g*, 20 min, 2×, and 16,000× *g*, 20 min); **4** **°C**	Concentration + SEC	**Vacuum filtration**; **centrifuged** (4000× *g*, 30 min); **Sepharose** **CL-2B**	
			* UC + SEC (+ F + DTT + PI)	**PI**; **SC**; **DTT**; **vortex**; **centrifuged** (16,000× *g*, 20 min); **filtered** (0.22 μm); **centrifuged** (120,000× *g*, 70 min); **Sepharose** **CL-2B**	
			PEG + SEC	**PEG solution** (4 °C, 12 h); **centrifuged** (1000× *g*, 30 min); **Sepharose** **CL-2B**	
[33]	Healthy (NS)	First-void, afternoon and evening urine	UC	**Centrifuged** (200,000× *g*, 60 min)	TEM NTA WB: CD63 and Hsp70
		**−80°C**; **SC** (2000× *g*, 30 and 60 min, 17,000× *g*)	* OUF (F + ExoQuick)	**Filtered** (0.22 µm); **centrifuged** (3000 g, 30 min); **dialysis** **membrane** (10,000 kDa MWCO); **ExoQuick**-**TC** (30 min, 4 °C); **centrifuged** (15,279 *g*, 2 min)	
[34]	Healthy (3)	First-void urine	Precipitation (ExoQuick std)	**ExoQuick-TC** (1:4 ratio, 4 °C, 12 h); **centrifuged** (1500× *g*, 30 min, 4 °C)	NTA WB: CD63 TEM
		**SC** (300× *g*, 10 min and 3000× *g*, 20 min, 4 °C and 17,000× *g*, 20 min, 4 °C); **DTT**; **filtered** (0.22 μm)	Precipitation (ExoQuick modified) (MEQ)	**ExoQuick-TC** (3:7 ratio, 4 °C, 12 h); **centrifuged** (10,000× *g*, 30 min, 4 °C)	
			Precipitation PEG6000 (PE6)	**PEG 6000** (4 °C, 12 h); **centrifuged** (4000× *g*, 60 min)	
			UC	**Centrifuged** (200,000× *g*, 75 min)	
			UF	**Amicon **Ultra-15Filter** (10 and 100 kDa MWCO); **centrifuged** (4000× *g*, 10 min)**	
			* SEC + MEQ or UF	**Concentrated** (qEV size exclusion columns)	
[35]	BHP (12)	Second-void urine	UC	**Centrifuged** (30 min, 2000× *g*, 4 °C and 45 min, 12,000× *g*, 4 °C); **centrifuged** (110,000× *g*, 2 h); filtered (0.22 µm); **centrifuged** (110,000× *g*, 70 min); **−80** **°C**	TEM NTA WB: Alix, CD9, Flotillin-1
		**Concentrated** (10kDa filter); **tris** **buffer**	Precipitation (ExoQuick)	**Concentrated** (10 kDa filter); **ExoQuick-TC** (4 °C, >12h); **centrifuged** **2×** (1500× *g*, 30 and 5 min)	
			SEC	**Concentrated** (10 kDa filter); **Sepharose** CL-2B;	
			* Bottom-up Optiprep (dUC)	**40**% **iodixanol**; **layered on bottom** of a discontinuous bottom-up ODG; **centrifuged** (18 h, 100,000× *g*, 4 °C); ODG fractions **collected** **from** **top**; **centrifuged** (3 h, 100,000× *g*, 4 °C); −80 °C	
			Top-down Optiprep (dUC)	**Concentrated** (10 kDa filter); **loaded** **on** **top** of a discontinuous top-down ODG; processed as above	

Main steps for uEV isolation are in bold. * Chosen isolation method. ^#^ Similar results were obtained, no particular method was chosen. Abbreviations: BHP—benign prostatic hyperplasia; BlCa—bladder cancer; DLS—dynamic light scattering; DTT—dithiothreitol; dUC—density gradient ultracentrifugation; D_2_O—deuterium oxide; ERB— exosome resuspension buffer; F—filtration; FSG—focal segmental glomerulosclerosis; IMN—idiopathic membranous nephropathy; IS—isolation solution; MEQ—ExoQuick modified; MWCO—molecular weight cut-off; NC—nanomembrane concentrators; NTA—nanoparticle tracking analysis; NS—non-specified; ODG—OptiPrep density gradient; OUF—optimized ultrafiltration; PEG—polyethylene glycol; PI—protease inhibitor; RT—room temperature; SC—serial centrifugation; SEC—size exclusion chromatography; STL—Solanum tuberosum (potato) lectin; std—standard; TEI—total exosome isolation; TEM—transmission electron microscopy; UC—ultracentrifugation; UF—ultrafiltration; WB—western blotting.

**Table 2:** uEV-derived protein and miRNA biomarker candidates in prostate cancer (PCa).

Study	Urine Source (*n*)/Type of Urine	Urine Pre-Treatment	Isolation Method	Characterization	Biomarker Candidates	Biomarker Performance			Biomarker Type
						AUC	SE (%)	SP (%)	
**Proteins**									
[66]	PCa (15) Healthy (15)	**SC** (15 min, 2000× *g*, RT and 30 min, 10,000× *g*)	**UC** **Centrifuged 2×** (100,000× *g*, 70 min, RT); **vortexed**; **filtered** (0.22 µm); **centrifuged** (100,000× *g*, 70 min)	DLS: Mean 149 nm TEM WB: CD9, CD63 and TSG101	↑TM256	0.87	--	--	Diagnosis
	Morning urine (PCa); first-void urine (healthy)				TM256 and LAMTOR1 combination	0.94	--	--	
[67]	mPCa (5) Healthy (13)	**SC** (400× *g* 7 min, 20 °C and 2000× *g*, 15 min); **vacuum** **filtered** (0.22 µm); **−80** **°C**	**UC** + **SEC** **Centrifuged** (400× *g* 7 min, 20 °C); **vacuum** **filtered** (0.22 µm); **centrifuged** (200,000× *g*, 2 h, 4 °C); **Sepharose** **CL-2B**	NTA: Mean 118 nm, peak 73 nm cryo-EM: ~100 nm ELISA: CD9, ApoB, THP, HAS WB: TSG101, ALIX, LAMP2, HAS	↑Afamin, cardiotrophin-1, CDON, ARTS-1, FGF19, IL17RC, NAMPT, IL1RAPL2, CD226, IGFBP2, CCL16, TNFSF18, IGFBP5; AADC	--	--	--	mPCa predictive treatment (prognosis)
	Morning urine (excluding first-void)								
[68]	PCa (low- and high-grade) (18) Negative biopsy (11)	**Centrifuged** (2000× *g*, 30 min); −80 °C	**UC** **Centrifuged** (17,000× *g*, 30 min); **centrifuged** **2×** (100,000× *g*, 90 min); **DTT**; **centrifuged** (100,000× *g*, 90 min); **−80** **°C**; **MPEX** **PTS** **solution** (95 °C, 5 min); **centrifuged** (100,000× *g*, 30 min, 4 °C)	TEM WB: CD9	↑ FABP5 and significantly associated with GS	0.76 (GS ≥ 6)	--	--	High-GS PCa
	First catch urine after DRE					0.86 (GS ≥ 7)	60.0	100	
[69]	PCa (53) -Low-grade PCa -High-grade PCa Negative biopsy (54)	**Centrifuged** (2500× *g*, 10 min, 4 °C); PI; −80 °C	**UC** **Centrifuged** (16,500× *g*, 20 min); **DTT**; **centrifuged** (16,500× *g*, 20 min); **filtered** (0.2 μM); **centrifuged** (100,000× *g*, 120 min, 4 °C); **centrifuged** (100,000× *g*, 60 min, 4 °C)	TEM NTA WB: TSG101, CD81 or Rab5	ADSV-TGM4 combination classifies benign and PCa.	0.65	--	--	Diagnosis and prognosis
					CD63-GLPK5-SPHMPSA-PAPP combination distinguish between high- and low-grade PCa	0.70	--	--	
	First catch urine after DRE								
[70]	PCa (26) Healthy (16) Morning urine	**Centrifuged** (15 min, RT, 2000× *g*)	**UC** **Centrifuged** (30 min, 10,000× *g*); **centrifuged** (100,000× *g*, 70min, RT); **centrifuged** (100,000× *g*, 70 min, 4 °C); **filtered** (0.22 µm); **centrifuged** (100,000× *g*, 70 min, 4 °C)	NP	↑Flotillin 2 (WB)	0.91	88	94	Diagnosis
	(PCa); first-void urine (healthy)				↑Flotillin 2, Parkinson protein 7 combination (ELISA)	--	68	93	
[35]	PCa (12) Prior to and three months after local treatment Healthy (12)	**Concentrated** (10 kDa filter device); **tris** **buffer**	**Bottom-up Optiprep** (**dUC**) **40**% **iodixanol**; **layered on bottom** of a discontinuous bottom-up ODG; **centrifuged** (18 h, 100,000× *g*, 4 °C); ODG fractions **collected** **from** **top**; **centrifuged** (3 h, 100,000× *g*, 4 °C); **−80** **°C**	TEM NTA: Mean 132 nm, peak 111 nm	↑FKBP5, FAM129A, RAB27A, FASN, NEFH	--	--	--	Diagnosis
	Second-void urine								
**miRNAs**									
[71]	PCa (48) Negative biopsy (26)	−**80** **°C**; **centrifuged** (20,000× *g*, 30 min, 4 °C)	**dUC** **Centrifuged 2×** (100,000× *g*, 90 min, 4 °C); **30**–**40**% **sucrose** **gradient**; **centrifuged** (100,000× *g*, 90 min)	TEM: 50–150 nm WB: TSG101 and ALIX	isomiR panel: ↑ miR-204; ↓ miR-21 and miR-375	0.82	--	--	PCa diagnosis
	First catch urine after DRE								
[72]	PCa (14) Healthy (20)	**SC** (400× *g*, 20 °C, 20 min and 17,000× *g*, 20 °C, 20 min); **−20** **°C**	**UC** **Centrifuged** (100,000× *g*, 18 °C, 90 min); **filtered** (0.1 μm); **centrifuged** (100,000× *g*, 18 °C, 90 min)	TEM: 20–230 nm Immunogold staining: CD63, CD9 and CD24	↑ miR-19b	--	93	100	PCa diagnosis
	NS								
[73]	PCa (60) BPH (37) Healthy (24)	**Centrifuged** (2000× *g*, 30 min, 4 °C); **PI**; **−80** **°C**	**UC** **Centrifuged** (17,000× *g*, 4 °C, 10 min); **IS**; **DTT**; **centrifuged** (17,000× *g*, 4 °C, 10 min); **centrifuged** 2× (200,000× *g*, 4 °C, 60 min) **HFD** **Separating** **funnel** connected with **dialysis** **membrane** (1000 kDa MWCO)	TEM NTA: <300 nm WB: TSG101, CD63, CD9, and ALIX	↑ miR-145 Compared with BPH and healthy controls. In GS ≥ 8 tumors compared with GS ≤ 7	0.623	--	--	PCa diagnosis and prognosis
	First-void urine								
[74]	PCa (90) BPH (10) Untreated BlCa (60) Healthy (50)	**PI**; **−80°C**	**ExiqonmiRCURYexosomeisolationkit**	SEM: ~100 nm WB: CD63	miR-2909: ↑with severity Distinguish PCa from BlCa.	--	--	--	PCa diagnosis and prognosis
	NS								
[75]	PCa (28) Healthy (19)	**Centrifuged** (2000× *g*, 15 min, RT); **centrifuged** (10,000× *g*, 30 min, RT)	**UC** **Centrifuged** (100,000× *g*, 70 min, RT); **centrifuged** (100,000× *g*, 70 min, 4 °C); **filtered** (0.22 µm); **centrifuged** (100,000× *g*, 70 min, 4 °C); **−80** **°C**	NP	↓ miR-196a-5p ↓ miR-501-3p	0.92 (NGS)	100	89	PCa diagnosis
	NS					0.72	--	--	
[76]	PCa (52) Healthy (10)	**SC** (2000× *g*, 20 min, 4 °C and 2000× *g*, 5 min, 4 °C); **QIAzol**; **−80** **°C**	**UC** **Centrifuged** (17,000× *g*, 45 min, 4 °C); **centrifuged** (200,000× *g*, 2 h, 4 °C); **−80** **°C**	TEM	↑ miR-21, miR-375 and let-7C	0.71; 0.80; 0.68	--	--	PCa diagnosis and prognosis
	Freshly voided, collected after massage								
[77]	PCa (10) BPH (10) Healthy (10)	**Centrifuged** (17,000× *g*, 20 °C, 20 min); **−20** **°C**	**UC** **Centrifuged** (100,000× *g*, 18 °C, 90 min); **filtered** (0.1 μm); **centrifuged** (100,000× *g*, 18 °C, 90 min)	NP	5 miRNA pairs (miR-30a: miR-125b; miR-425: miR-331; miR-29b: miR-21; miR-191: miR-200a; miR-331: miR-106b)	97.5% accuracy.	--	100	PCa diagnosis
	NS								
[78]	PCa (28) Negative biopsy (28)	**SC** (650× *g*, 10 min, RT and 10,000× *g*, 30 min); **−80°C**	**Vn96****peptide** **Centrifuged** (17,000× *g*, 15 min, RT); **Vn96** **synthetic** **peptide** (30× *g*/ mL urine); **centrifuged** (17,000× *g*, 15 min, RT)	WB: CD9, CD63, CD24, Hsp/c70, PDCD6IP	↑ miR-375-3p and miR-574-3p panel	0.74	--	--	PCa diagnosis
	Freshly voided, collected after massage and DRE								

Isolation methods are in bold and underlined. Main steps for uEV isolation are in bold. ↑ Upregulated or ↓ downregulated in PCa. Abbreviations: AUC—area under the curve; BHP—benign prostate hyperplasia; DLS—dynamic light scattering; DRE—digital rectal examination; DTT—dithiothreitol; dUC—density gradient ultracentrifugation; EM—electron microscopy; GS—Gleason score; HAS—Hyaluronan synthases; HFD—hydrostatic filtration dialysis; HSP70—heat shock protein 70; IGFBP—insulin-like growth factor-binding protein; IS—isolation solution; mPCa—metastatic prostate cancer; MWCO—molecular weight cut-off; NP—not performed; NS—non-specified; NTA—nanoparticle tracking analysis; ODG—OptiPrep density gradient; PCa—prostate cancer; PI—protease inhibitor; RT—room temperature; SEC—size exclusion chromatography; SE—sensitivity; SP—specificity; STL—Solanum tuberosum (potato) lectin; TEI—total exosome isolation; TEM—transmission electron microscopy; THP—Tamm–Horsfall protein; UC—ultracentrifugation; UF—ultrafiltration; WB—western blotting.

**Table 3:** uEV-derived protein and miRNA biomarker candidates in bladder cancer (BlCa).

Study	Urine Source (*n*)/Type of Urine	Urine Pre-Treatment	Isolation Method(s)	Characterization	Biomarker Candidates	Biomarker Performance			Biomarker Type
						AUC	SE (%)	SP (%)	
**Protein**									
[85]	BlCa (2) Healthy (2)	**PMSF**; **centrifuged** (250× *g*, 10 min); **−80 °C**	**UC** **PI**; **Centrifuged** (250× *g*, 10 min) **centrifuged** (17,000× *g*, 30 min); **centrifuged** 2× (200,000× *g*, 60 min); **−80 °C**	NP	↑ Resistin, GTPase NRas, EPS8L2, Mucin 4, EPS8L1, RAI3, Alpha subunit of GsGTP binding protein, EHD4EH	--	--	--	BlCa diagnosis
	NS								
[86]	BlCa (28) Hernia (12)	**PI** and **sodium azide**; **centrifuged** (5000× *g*, 30 min, 4 °C); **−80 °C**	**UC** **Centrifuged** (17,000× *g*, 30 min, 4 °C); **centrifuged 2×** (100,000× *g*, 70 min, 4 °C)	TEM: 30–100 nm WB: TSG101 and CD9 Flow Cytometry: CD9	↑ TACSTD2	0.80	73.6	76.5	BlCa diagnosis and prognosis
	First-void urine								
[87]	BlCa (16) (high-, low-grade) Healthy (10)	**Centrifuged** (3500× *g*, 25 min, 4 °C); **filtered** (0.22 μm)	**UC** **Centrifuged 2×** (100,000× *g*, 4 °C, 1 h)	NTA TEM WB: ERM and CD9	↑ ApoB (high-grade)	--	--	--	BlCa diagnosis and prognosis
	First-void urine								
[88]	BlCa (129) Healthy (62)	**PI**; **centrifuged** (1000× *g*, 10 min); **−80 °C**	**UC** **Centrifuged** (17,000× *g*, 10 min, 4 °C); **IS**; **DTT**; **centrifuged** (17,000× *g*, 30 min, 4 °C); **centrifuged** (200,000× *g*, 1 h, 4 °C)	TEM: 50–100 nm WB: TSG101, Alix	↑ Alpha 1-antitrypsin	0.74	50.4	96.9	BlCa diagnosis and prognosis
	First-void urine				↑ Histone H2B1K	0.77	62.0	92.3	
[89]	BlCa pT1-pT3 (6) Healthy (6)	**SC** (400 g and 15,500× *g*); **−80 °C**	**UC** **Spun**; **centrifuged 2×** (200,000× *g*, 70 min); **centrifuged** (15,500× *g*)	TEM NTA: Mean 35–300 nm, peak 105 nm	↑ HEXB, S100A4, and SND1	--	--	--	BlCa diagnosis
	Perioperative (BlCa) NS (healthy)								
[60]	BlCa (10) Healthy (10)	**−80 °C**; **Centrifuged** (2000× *g*, 10 min); **filtered** (0.45 μm); **centrifuged** (30 min, 18,000× *g*)	**UC** **Centrifuged** (200,000× *g*, 16 h); **centrifuged** (200,000× *g*, 1 h); **filtered** (0.22 μm); **PI**	TEM NTA: peak ~124 nm Flow cytometry and WB: Alix TSG101, CD63, HSP70, Flotillin-1	↑ Mucin-1, CEACAM-5, EPS8L2, and moesin	--	--	--	BlCa Diagnosis
	First-void urine								
[90]	BlCa (13)	**Spun** (3000× *g*, 30 min); **filtered** (0.22 µm)	**UC** **Centrifuged** (100,000× *g*, 2 h); **−80 °C**	Flow cytometry: CD9 CD81, CD63 NTA: Peak ~155 nm TEM	↓ SLC4A1 ↑ TPP1, TMPRSS2, FOLR1, RALB and RAB35	--	--	--	BlCa recurrence
	Perioperative urine								
**miRNA**									
[91]	BlCa (16) Low-grade NMIBC to high-grade MIBC	**Centrifuged** (1200 and 2500 rpm, 20 min, 20 °C); **−80 °C**	**Norgen Urine Exosome RNA Isolation Kit**	NP	↑ miR-4454, miR-21, miR-205, miR-200C-3p, miR-29b-3p	--	--	--	BlCa diagnosis
	NS								
[92]	BlCa (TaG1, T1G3, ≥T2, CIS) (85) Healthy (35)	**Centrifuged** (3000 rpm, 4 °C, 15 min); **−20 °C**	**UC** **Centrifuged** (17,000× *g*, 10 min, 4 °C); **centrifuged** (200,000× *g*, 1 h, 4 °C); **DTT**; **centrifuged** (200,000 g, 1 h, 4 °C)	WB: Alix and TSG101	↑ miR-26a, miR-93, miR-191, and miR-940 panel	0.89	88	78	BlCa diagnosis
	NS								
[87]	BlCa (34) Low- and high-grade Healthy (9)	**Centrifuged** (3500× *g*, 25 min, 4 °C); **filtered** (0.22 μm)	**UC** **Centrifuged 2×** (1 h, 100,000× *g*, 4 °C)	WB: ERM and CD9 NTA TEM	↓ miR-375 in low-grade ↑ miR-146a high-grade	--	--	--	BlCa diagnosis and prognosis
	First-void urine								
[93]	BlCa (36) Healthy (24)	**SC** (2000× *g*, 30 min and 30 min, 17,000× *g*)	**UC** **Centrifuged 2×** (130,000× *g*, 90 min); **−80 °C**	NTA: <200 nm TEM ELISA: CD9 WB: CD9 and CD63	↑ miR-21-5p	0.90	75.0	95.8	BlCa diagnosis
	Voided urine (NS)				↑ miR-155-5p, miR-15a-5p, miR-132-3p, miR-31-5p	0.820; 0.841; 0.821; 0.821	--	--	
[94]	NMIBC (17) MIBC (20)	**−80 °C**; **SC** (2000× *g*, 4 °C, 20 min and 15,000× *g*, 30 min)	**Total Exosome Isolation From Urine Kit**	WB: CD63 and GM130 NTA: Median ~125 nm TEM: ~48 nm	↑ miR-146b-5p and miR-155-5p in MIBC than NMIBC	--	--	--	MIBC prognosis
	Perioperative urine								

Isolation methods are in bold and underlined. Main steps for uEV isolation are in bold. ↑ Upregulated or ↓ downregulated in BlCa Abbreviations: AUC—area under the curve; BlCa—bladder cancer; DTT—dithiothreitol; IHC—immunochemistry; IS—isolation solution; MIBC—muscle invasive bladder cancer; NMIBC—non muscle invasive bladder cancer; NP—not performed; NS—non-specified; NTA—nanoparticle tracking analysis; PI—protease inhibitor; PMSF—phenylmethanesulfonylfluoride; SE—sensitivity; SP—specificity; TEM—transmission electron microscopy; THP—Tamm–Horsfall protein; UC—ultracentrifugation; WB—western blotting.

**Table 4:** uEV-derived protein and miRNA biomarker candidates in renal cell carcinoma (RCC).

Study	Urine Source (*n*)/ Type of Urine	Urine Pre-Treatment	Isolation Method(s)	Characterization	Biomarker Candidates	Biomarker Performance			Biomarker Type
						AUC	SE (%)	SP (%)	
**Proteins**									
[104]	RCC (29) Healthy (23)	**–80 °C**; **centrifuged** (10 min, 4 °C, 1000× *g*); **PI**; **centrifuged** (15 min, 4 °C, 17,000× *g*)	**OptiPrep (dUC)** Discontinuous **OptiPrep gradient**; **centrifuged** (100,000× *g*, 16 h) collected from **top** of gradient; **centrifuged** (100,000× *g*, 3 h)	TEM WB: CD9, TSG101 and Flotillin-1	↑ MMP-9, CP, PODXL, DKK4 and CAIX	0.938; 1; 1; 0.979; 0.862	--	--	RCC diagnosis
	Second morning urine samples				↓ AQP1, EMMPRIN, CD10, Dipeptidase 1 and Syntenin-1	0.891; 0.879, 0.794; 0.760; 0,733	--	--	
**miRNAs**									
[105]	ccRCC (81) Benign kidney tumors (24) Healthy (33)	**Centrifuged** (2000× *g*, 10 min, 4 °C); **−80 °C**	**Norgen kit**	NP	Combinations of miR-126-3p with miR-449a or miR-34b-5p distinguish ccRCC from controls.	0.84; 0.79	--	--	RCC diagnosis
	Preoperative urine				Combination of miR-126-3p and miR-34b-5p distinguish SRM from controls.	0.79	--	--	
					miR-126-3p and miR-486-5p combination differentiate benign lesions from ccRCC	0.85	--	--	
[106]	ccRCC (T1aN0M0) (70) Healthy (30)	**Centrifuged** (2000× *g*, 5 min, 4 °C); **filtered** (0.22 μm); **−80 °C**	**UC** **Centrifuged** (150,000× *g*, overnight, 4 °C); **centrifuged** (150,000× *g*, 4 °C, 2 h)	TEM NTA: Peak ~116 nm WB: CD81, CD63, CD9	↓ miR-30C-5p	0.82	68.57	100	RCC diagnosis
	Morning urine (NS)								

Isolation methods are in bold and underlined. Main steps for uEV isolation are in bold. ↑ Upregulated or ↓ downregulated in RCC. Abbreviations: AUC—area under the curve; ccRCC—clear cell renal cell carcinoma; dUC—density gradient ultracentrifugation; NP—not performed; PI—protease inhibitors; RCC—renal cell carcinoma; SE—sensitivity; SP—specificity; SRM—small renal masses; TEM—transmission electron microscopy; UC—ultracentrifugation; WB—western blotting.
